# Supplementary material for: Prediction of Post Traumatic Epilepsy Using MR‐Based Imaging Markers
Source: Hum Brain Mapp. 2024 Nov 19;45(17):e70075. doi: 10.1002/hbm.70075 (PMC11574740; doi:10.1002/hbm.70075)
Supplement: Supplementary file 1 — Data S1. [file HBM-45-e70075-s001.pdf]

Supplemental Material

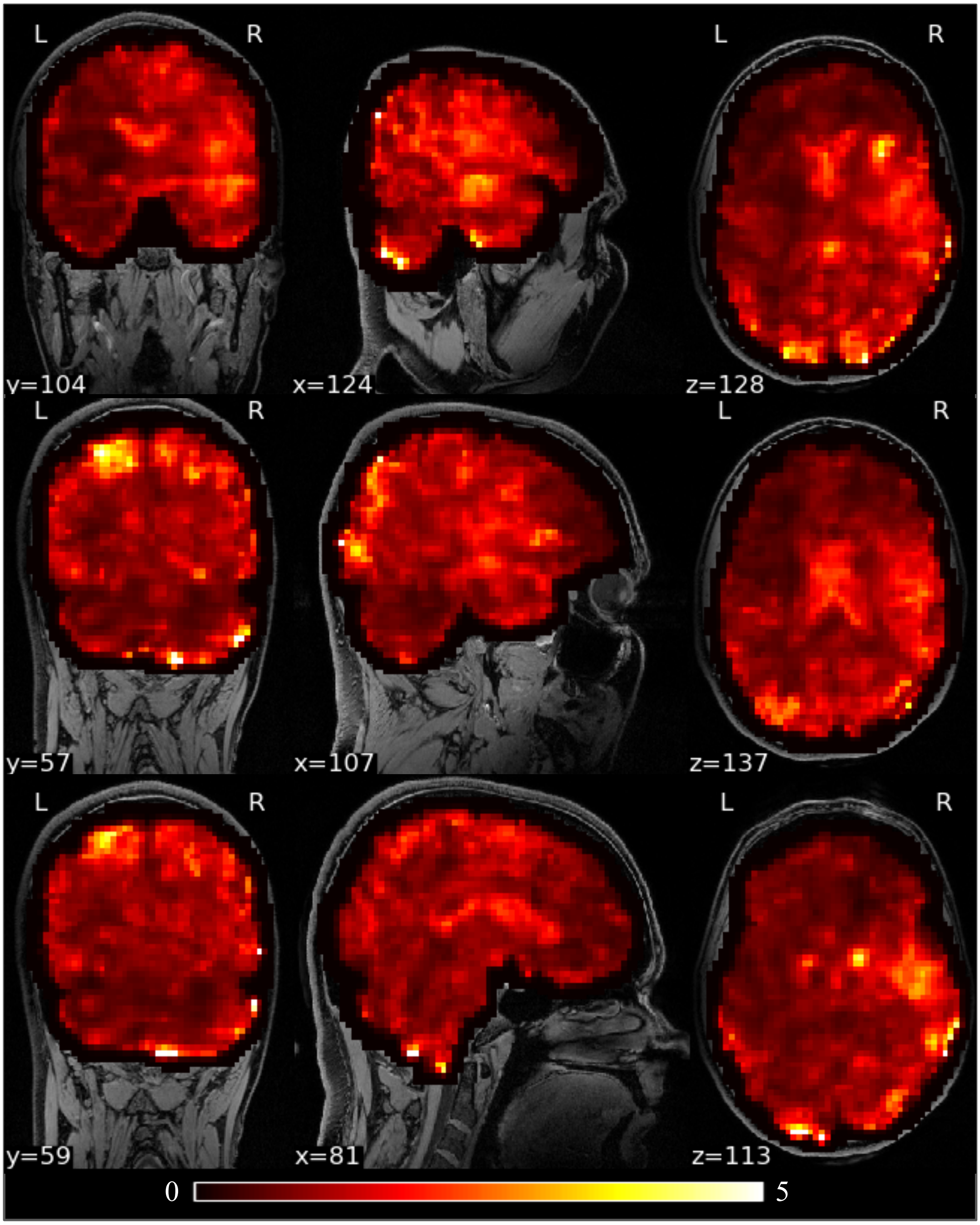

FIGURE S1 Voxel-based PTE vs. non-PTE group comparison of lesion maps overlaid on the USCBrain atlas. The color code depicts f-values, shown in a region where p-value < 0.05, resulting from the F-test (with permutations). Prominent significant clusters are located in the left temporal lobe, bilateral occipital lobe, cerebellum, and right parietal lobe.

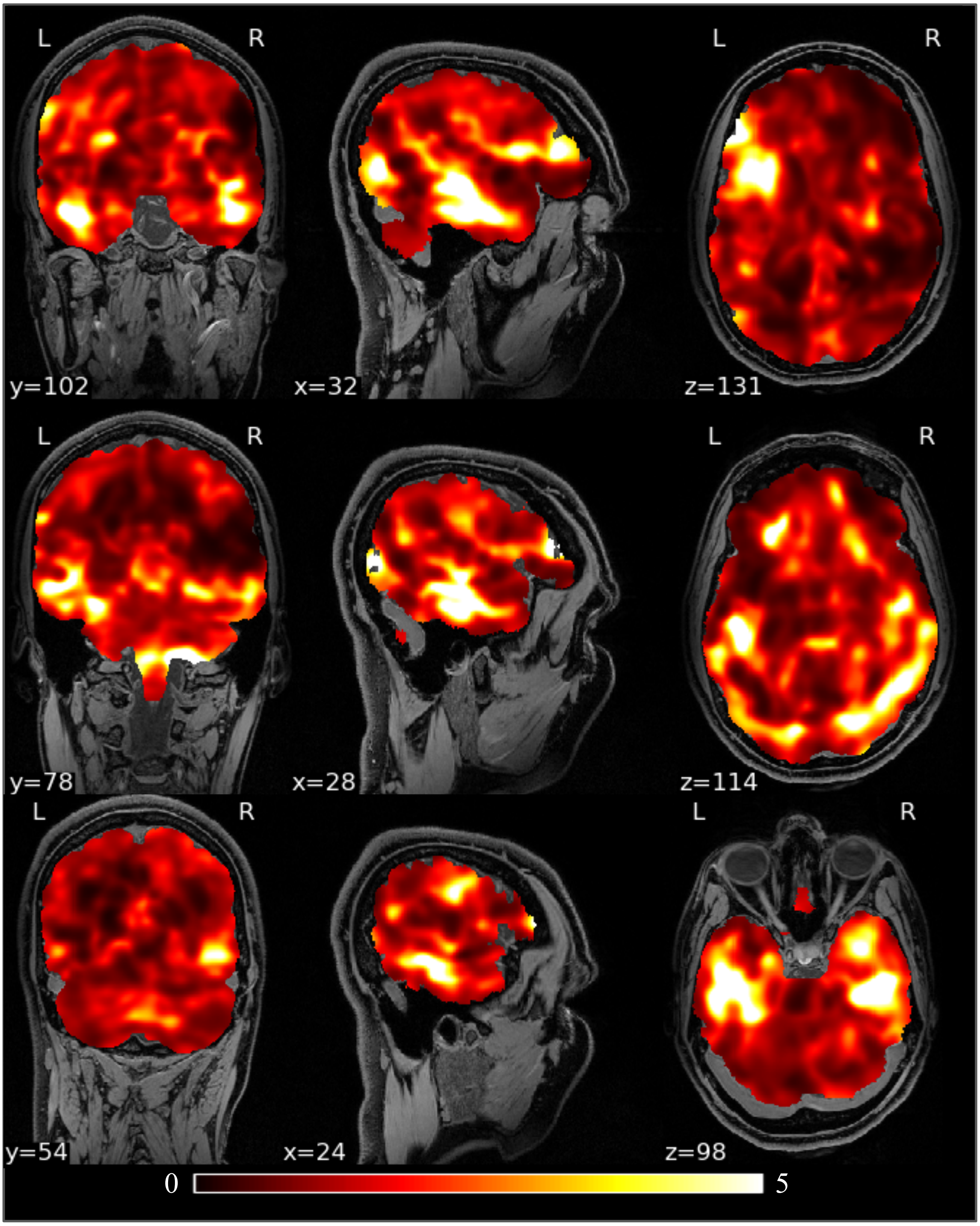

FIGURE S2 Differences in ALFF between the PTE and non-PTE groups. The results are color-coded f-statistic thresholded by FDR corrected p-values ($p < 0.05$) derived using a permutation test. Significant clusters are visible in the left temporal lobe, bilateral occipital lobes, cerebellum, and right parietal lobe. The higher F-values in lesion compared to ALFF comparisons show that lesion could be a stronger marker of PTE compared to functional changes at an early stage after TBI.

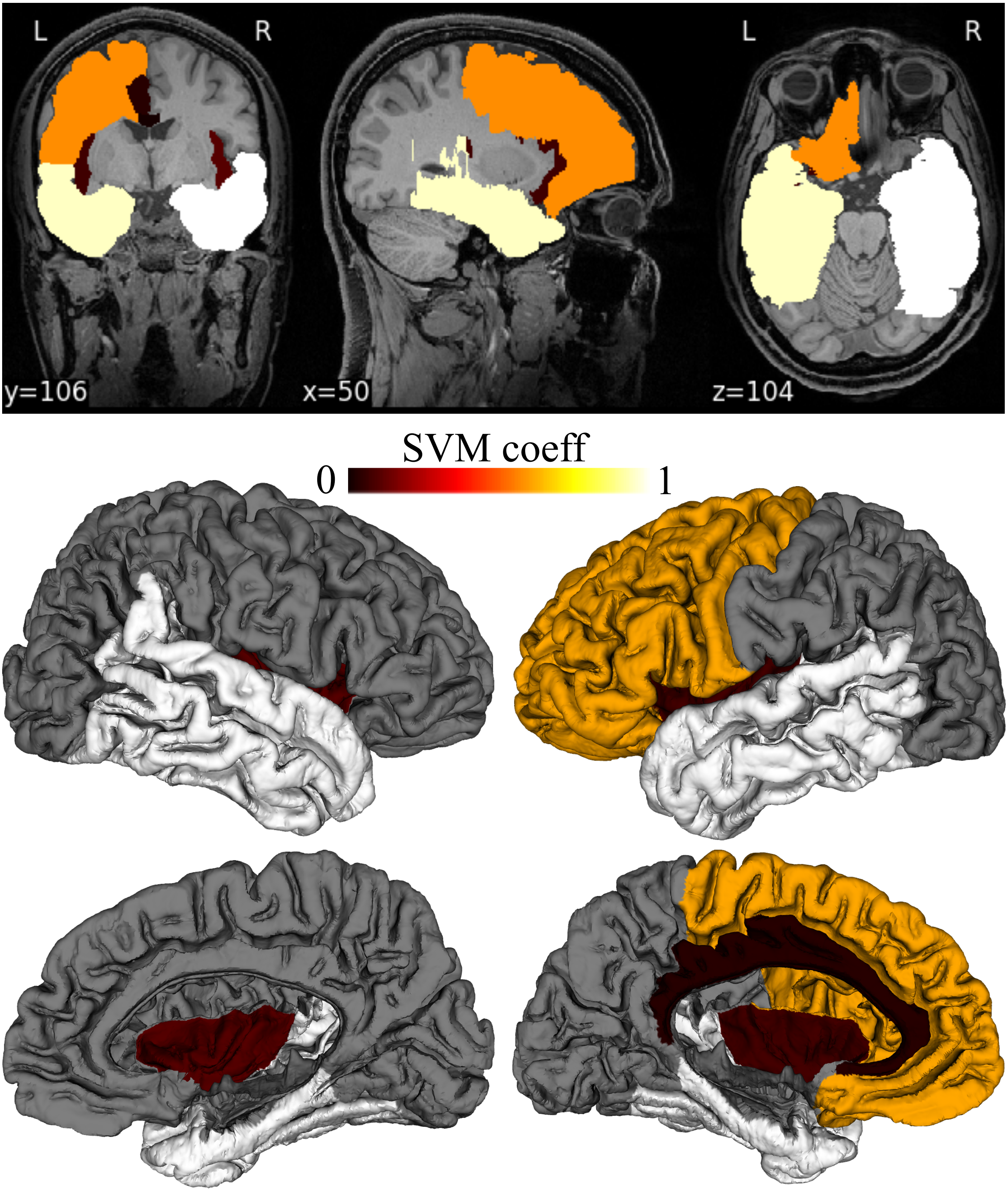

FIGURE S3 The feature importance map is shown as color-coded ROIs overlaid on the USCBrain atlas. Both cortical surface and volumetric ROIs are shown.

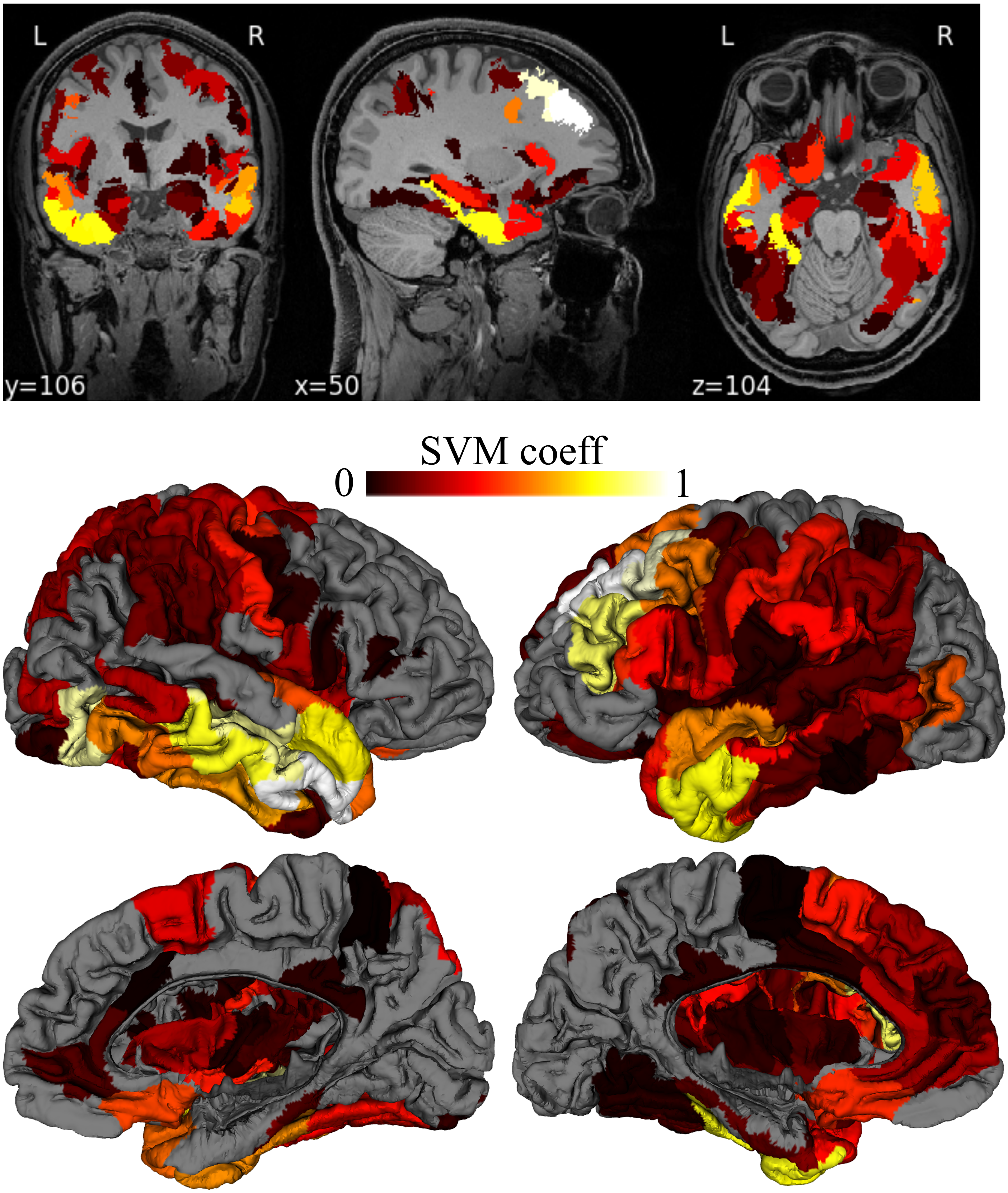

FIGURE S4 The feature importance map is shown as color-coded ROIs overlaid on the Brainnetome atlas. Both cortical surface and volumetric ROIs are shown.
