# Supplementary figures and images for: Prediction of Post Traumatic Epilepsy Using MR‐Based Imaging Markers
Source: Hum Brain Mapp. 2024 Nov 19;45(17):e70075. doi: 10.1002/hbm.70075 (PMC11574740; doi:10.1002/hbm.70075)

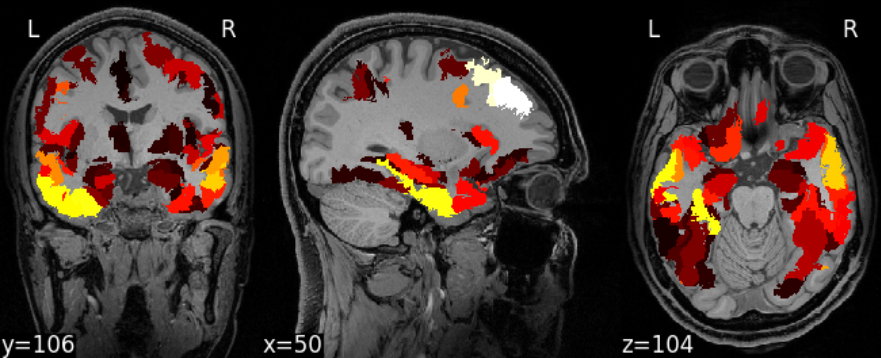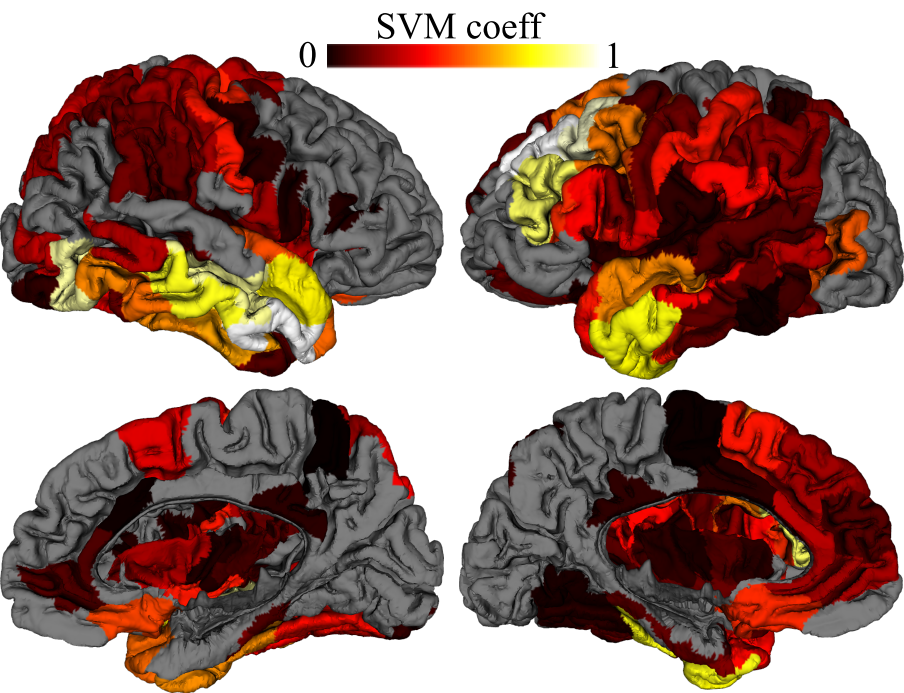

Supplement: Supplementary file 2 — Data S2. [file HBM-45-e70075-s002.zip › supp_material/feat_imp_brainnetome.pdf]

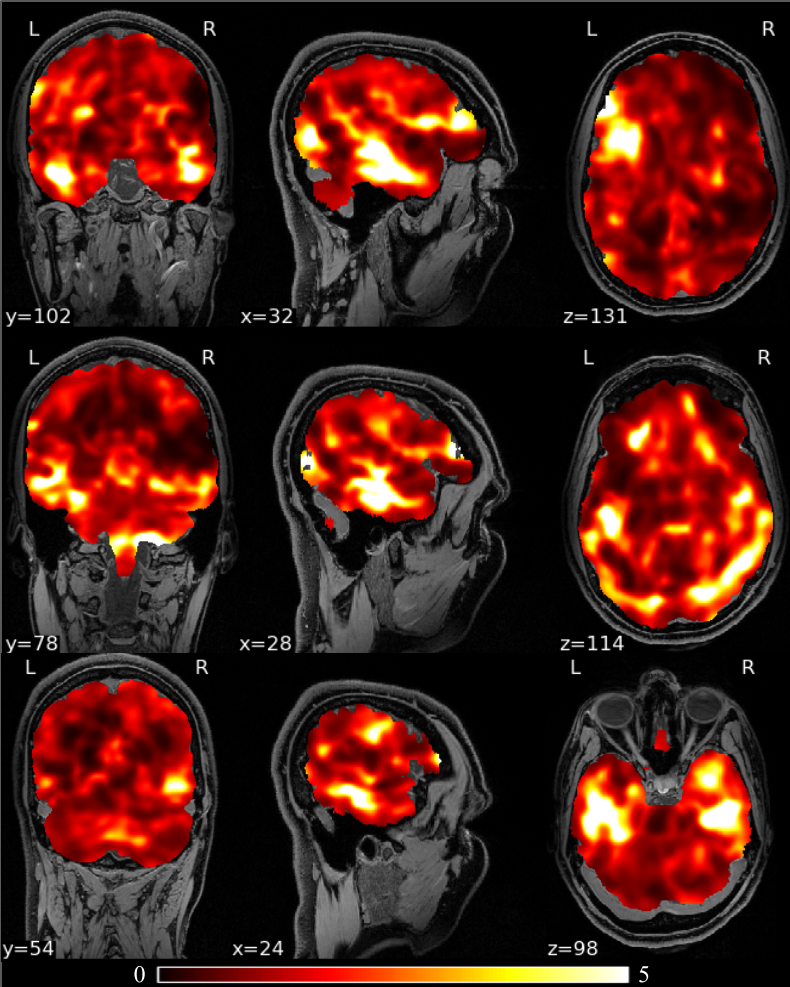

Supplement: Supplementary file 2 — Data S2. [file HBM-45-e70075-s002.zip › supp_material/fval_alff_unthresholded.pdf]

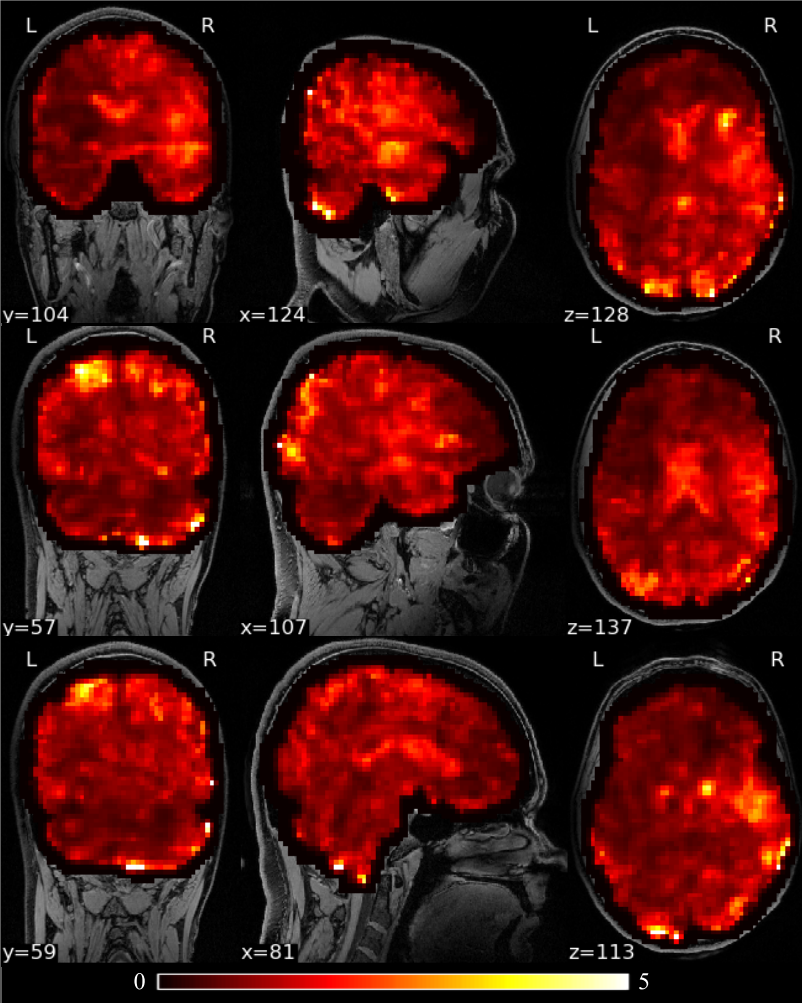

Supplement: Supplementary file 2 — Data S2. [file HBM-45-e70075-s002.zip › supp_material/fval_lesion_unthresholded.pdf]

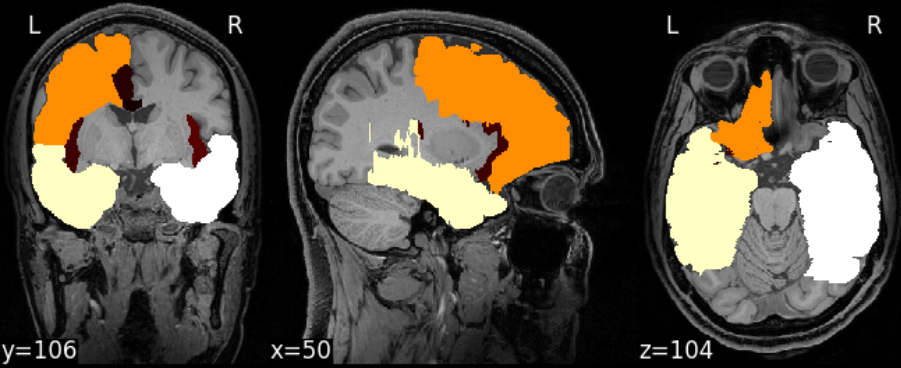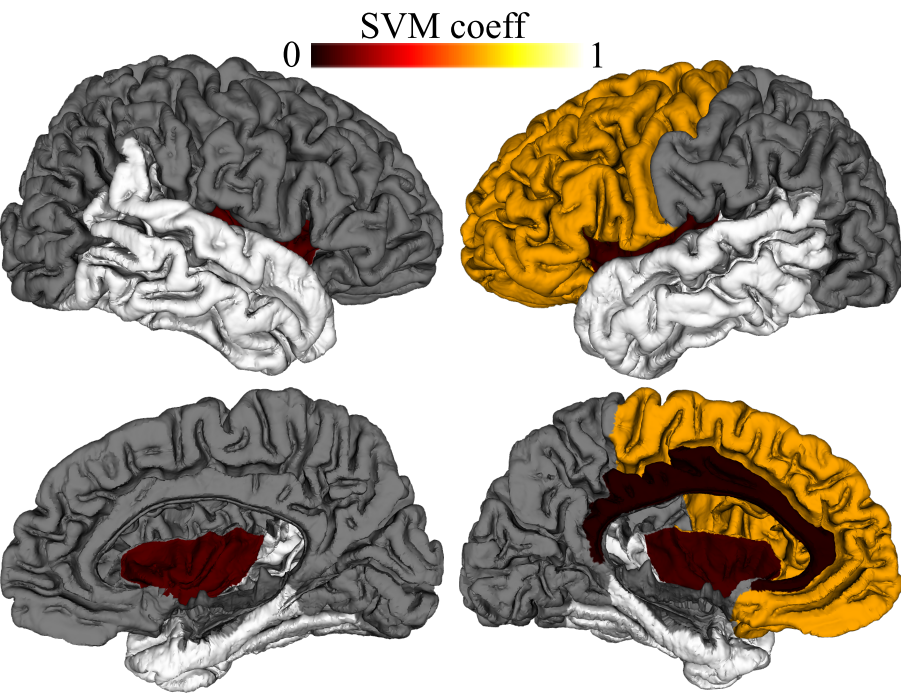

Supplement: Supplementary file 2 — Data S2. [file HBM-45-e70075-s002.zip › supp_material/feat_imp_usclobes.pdf]
